# Alberta Spinal Muscular Atrophy Newborn Screening—Results from Year 1 Pilot Project

**DOI:** 10.3390/ijns9030042

**Published:** 2023-07-27

**Authors:** Farshad Niri, Jessie Nicholls, Kelly Baptista Wyatt, Christine Walker, Tiffany Price, Rhonda Kelln, Stacey Hume, Jillian Parboosingh, Margaret Lilley, Hanna Kolski, Ross Ridsdale, Andrew Muranyi, Jean K. Mah, Dennis E. Bulman

**Affiliations:** 1 Alberta Newborn Screening Laboratory, Alberta Precision Laboratories, Edmonton, AB T6G 2H7, Canada; 2 Department of Medical Genetics, University of Alberta, Edmonton, AB T6G 2H7, Canada; 3 Department of Pediatrics, Cumming School of Medicine, University of Calgary, Calgary, AB T3B 6A8, Canada; 4 Department of Pathology and Laboratory Medicine, University of British Colombia, Vancouver, BC V6H 3N1, Canada; 5 Department of Medical Genetics, University of Calgary, Calgary, AB T2N 4N2, Canada; 6 Department of Pediatrics, University of Alberta, Edmonton, AB T6G 1C9, Canada

**Keywords:** SMA, newborn screening, *SMN1*, multiplex qPCR, gene therapy

## Abstract

Spinal muscular atrophy (SMA) is a progressive neuromuscular disease caused by biallelic pathogenic/likely pathogenic variants of the *survival motor neuron 1* (*SMN1*) gene. Early diagnosis via newborn screening (NBS) and pre-symptomatic treatment are essential to optimize health outcomes for affected individuals. We developed a multiplex quantitative polymerase chain reaction (qPCR) assay using dried blood spot (DBS) samples for the detection of homozygous absence of exon 7 of the *SMN1* gene. Newborns who screened positive were seen urgently for clinical evaluation. Confirmatory testing by multiplex ligation-dependent probe amplification (MLPA) revealed *SMN1* and *SMN2* gene copy numbers. Six newborns had abnormal screen results among 47,005 newborns screened during the first year and five were subsequently confirmed to have SMA. Four of the infants received *SMN1* gene replacement therapy under 30 days of age. One infant received an *SMN2* splicing modulator due to high maternally transferred AAV9 neutralizing antibodies (NAb), followed by gene therapy at 3 months of age when the NAb returned negative in the infant. Early data show that all five infants made excellent developmental progress. Based on one year of data, the incidence of SMA in Alberta was estimated to be 1 per 9401 live births.

## 1. Introduction

Spinal muscular atrophy (SMA) is an autosomal recessive disorder characterized by progressive muscle weakness and atrophy of limb, trunk, bulbar, and respiratory muscles, which results in feeding and respiratory difficulties [1]. The onset of the disorder varies and ranges from prenatal period to adulthood. It is caused by loss of function (LOF) variants in the *SMN1* gene [2]. The majority (~96%) of SMA patients have homozygous absence of exon 7 or both exons 7 and 8 in *SMN1* as a result of deletion or gene conversion with the highly homologous nearby *SMN2* gene [3]. The remaining 4% of SMA cases are compound heterozygous for a loss of function point mutation in one *SMN1* allele and a deletion or gene conversion in the other, or very rarely, biallelic *SMN1* point mutations. Some individuals with SMA have an additional (>2) copies of *SMN2* [4]. The presence of extra copies of *SMN2* can partially compensate for the deficiency in *SMN1* resulting in milder phenotype and later ages of onset [5,6]. In patients with milder forms of the disease (SMA types 2, 3, and 4), gene conversion, in which *SMN1* exon 7 is replaced by *SMN2* exon 7, is often the cause of the disease instead of deletions of *SMN1* [3,7]. These mutations result in reduced expression but not complete loss of survival motor neuron (SMN) protein, which is involved in the maintenance of the homeostatic environment of motor neurons [8]. Lack of *SMN* leads to degeneration of the anterior horn cells in the spinal cord and brainstem [1].

Prior to 2016, SMA management mainly focused on managing symptoms and providing supportive care. However, since then, effective disease-modifying therapies (DMTs) have shown great potential in halting disease progression. Currently, there are three SMA-specific DMTs that have been approved by the United States Food and Drug Administration (FDA) and Health Canada [9,10,11,12,13,14,15,16,17]. Nusinersen (Spinraza^®^) is an antisense oligonucleotide that modifies *SMN2* splicing when administered intrathecally; it was approved by the FDA in 2016 and by the European Medicines Agency (EMA) in 2017 for all subtypes of SMA. In July 2019, the FDA approved onasemnogene abeparvovec-xioi (Zolgensma^®^), a viral-mediated *SMN1* gene replacement therapy, for SMA in children under the age of 2 years, and the EMA approved it in May 2020 for SMA patients who have two or three copies of the *SMN2* gene. Risdiplam (Evrysdi^®^), an oral *SMN2* splicing modifier, was approved by the FDA in July 2020 for SMA patients who are two months of age or older [18]. All three DMTs were also approved in the province by Alberta Health in 2018, 2021, and 2022, respectively.

Clinical research studies have shown that early treatment is the most effective, with the best outcomes observed among those who were treated before onset of symptoms [9,13,19]. Early identification of infants with SMA is made possible by newborn screening (NBS), which enables prompt referral for those who test positive to receive confirmatory diagnostic testing and implementation of treatment plans. Therefore, there has been a recent emphasis on incorporating SMA into NBS programs, making it more crucial than ever to develop NBS techniques specific to SMA. In 2018, SMA was added to the recommended screening panel in the United States and has been implemented in most states since then. In Canada, NBS for SMA was started in Ontario in 2020 and has subsequently become available in all provinces and territories except Quebec and Nova Scotia, where it is planned [20]. Testing became available in Alberta in February 2022.

Here, we present the development and validation of a quantitative polymerase chain reaction (qPCR) based SMA assay, capable of identifying the homozygous absence of exon 7 of the *SMN1* gene, and suitable for use in SMA NBS. The SMA test was combined with the already established severe combined immunodeficiency (SCID) qPCR assay, making it a multiplex test. This allowed for a smooth transition with no need for extra blood samples and minimal added cost for testing. Additionally, we further evaluated the performance of the assay by analyzing the results from screening nearly 50,000 newborns in the first-year pilot project.

## 2. Materials and Methods

The Alberta SMA NBS pilot project was launched on 28 February 2022 using a multiplex qPCR screening assay and DNA extracted from dried blood spots (DBS) cards to detect the absence of *SMN1* exon 7. DNA extracted from a second blood sample using multiplex ligation-dependent probe amplification (MLPA) confirms the diagnosis and determines the *SMN2* copy number. This study was approved by the Conjoint Health Research Ethics Board at the University of Calgary.

### 2.1. Patients and Samples

#### 2.1.1. Validation Study

A total of 3200 DNA samples, isolated from de-identified residual putative normal NBS specimens from 2016, were used to evaluate the performances of the multiplex assay and to establish the cut-offs. Preliminary cut-offs were established based on this population. The assay performance and its potential clinical application was further evaluated by testing DBS reference samples with known copy numbers of the *SMN1* and *SMN2* genes, archived, and donated DBS specimens with written consent obtained from patients with confirmed diagnosis of SMA.

#### 2.1.2. Alberta Pilot Project February 2022–February 2023

Data from 47,005 samples were processed as part of routine NBS program for SCID/SMA between 28 February 2022 and 27 February 2023.

### 2.2. DBS Punching and DNA Isolation

The DBS punching was performed using a Wallac DBS Puncher (PerkinElmer, Waltham, MA, USA) into wells of 96-well plates. The 3.2 mm diameter punches underwent semi-automated DNA extraction in a Tecan Robotic Liquid Handler (Tecan, US, Morrisville, NC, USA) using Generation DNA Purification and Elution Solutions (QIAGEN, Germantown, MD, USA). The extraction protocol included two purification washes of the DBS by adding 100 µL of Generation DNA Purification solution per well, followed by one wash with Generation DNA Elution Solution. All washes were performed at 37 °C for 10 min on a microplate shaker. After discarding the Elution buffer, 75 µL of new Elution solution was added to each well and incubated for 30 min at 98 °C while being shaken in a VorTemp 56 (Labnet, Edison, NJ, USA) at 1300 rpm. Plates were then cooled down to ambient temperature.

### 2.3. SMN1 Multiplex qPCR Assay

The *SMN1* multiplex assay was performed on a QuantStudio Real-Time PCR System (ThermoFisher Scientific, Waltham, MA, USA). The *SMN1* assay was designed using the primer sets published by Baker et al., with some modifications [21]. In the multiplex qPCR reaction, DNA extracted from DBS was used to amplify an approximately 140 bp region targeting the *SMN1* c.840C nucleotide. The 15 μL reaction contained 6 μL of the extracted DNA, 7.5 μL of Tough Mix (Quantabio, Beverly, MA, USA), 0.1 μM of the *SMN1*, *RPP30*, and TREC forward and reverse primers, ABY-labeled *SMN1* probe, *SMN2* blocker, VIC-labeled *RPP30* probe, and 0.25 μM of FAM-labeled TREC probe (ThermoFisher custom assays) (Supplementary Table S1). PCR condition was 95 °C for 10 min, followed by 45 cycles of 95 °C for 15 s, 60 °C for 30 s. *RPP30* was used as the internal control. Cycle threshold levels were manually determined to be 0.04 by an inspection of the amplification curves. CT values were reported by the instrument software.

### 2.4. Validation Study

The multiplex qPCR assay performance was evaluated by testing 3200 DNA samples isolated from de-identified residual NBS specimens from 2016, and preliminary cut-offs for *SMN1* and the internal control (*RPP30*) were established based on these samples. The assay performance and its potential clinical application was further evaluated by testing DBS reference samples with known copy numbers of the *SMN1* and *SMN2* genes, archived and donated DBS specimens with confirmed diagnosis of SMA. The assay’s performance was assessed by determining assay sensitivity and specificity, repeatability, intermediate precision, and reproducibility. Well-to-well carryover between punches using just filter blanks was also assessed. Due to the inaccurate yields of TREC analyte in old DBS samples from 2016, TREC multiples of the median (MoM) values for clinical use could not be determined during the SMA validation study. TREC values were subsequently determined during the Alberta Pilot project using fresh samples.

### 2.5. Alberta Pilot Project

Data from 47,005 newborns were processed during routine NBS between 28 February 2022, and 27 February 2023. The multiplex qPCR assay was performed as described above, data were collected and analyzed to provide TREC MoM values and assess for homozygous absence of exon 7 in the *SMN1* gene.

### 2.6. MLPA

Blood was collected on all patients who showed homozygous absence of *SMN1* for diagnostic confirmatory testing using the SALSA^®^ MLPA^®^ Probemix P021 SMA kit (MRC Holland, Amsterdam, The Netherlands) following the manufacturer’s protocol. Analysis was performed using the SEQUENCE Pilot module MLPA^®^ version 4.4.0 (JSI medical systems GmbH, Ettenheim, Germany). Copy numbers of both *SMN1* and *SMN2* genes were reported.

## 3. Results

**Figure 1:**
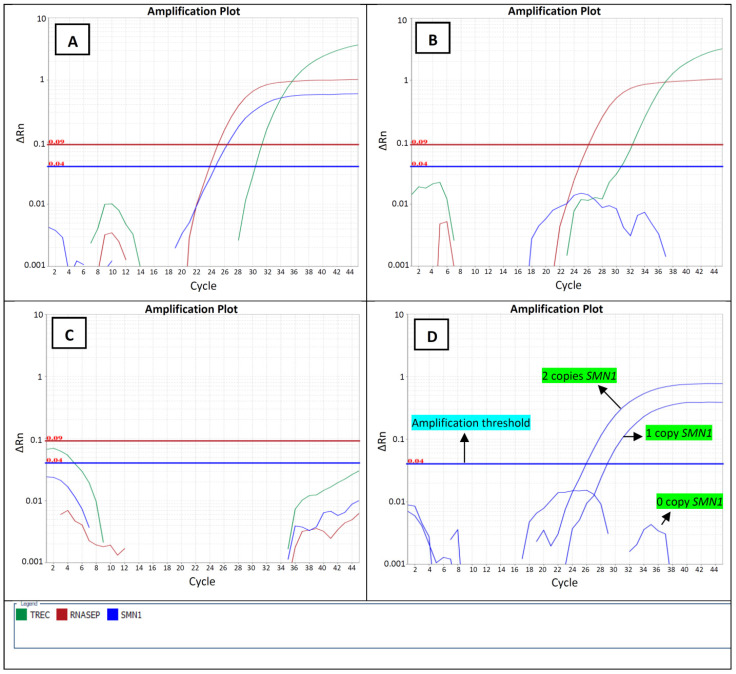
Representative Amplification Plots Generated using Taqman Probes and the CT Method of Relative Quantification Developed for Newborn Screening of SMA/SCID. Plots are shown for a normal sample (**A**), a sample with a positive SMA screen (**B**), and a no-template control (**C**). Panel (**D**) compares the *SMN1* amplification curves in a heterozygous deletion, a homozygous deletion, and a normal sample. No *SMN1* amplification was detected in the positive SMA screen as expected. Plots show the CT value of three targets (blue: SMN1, red: *RPP30* and green: TREC). ΔRn = the reporter signal normalized to the fluorescence signal of Applied Biosystems ROX Dye minus the baseline; ΔRn is plotted against PCR cycle number. Amplification threshold level was manually determined to be 0.04 for *SMN1*.

The SMA test was developed and combined with the previously established SCID qPCR assay to be implanted in the Alberta Newborn Screening program to screen for SMA. The assay also contained the *RPP30* assay as the internal control for quality purposes. Samples from patients with SMA did not show any amplification, while samples with one or more *SMN1* copy numbers showed a characteristic successful amplification curve, as expected (Figure 1). There was a clear distinction between the CT value of samples with 0 copies of *SMN1* and samples with ≥1 copy of *SMN1*, regardless of the *SMN2* copy number.

### 3.1. Determination of the Cut-Offs

To determine reference ranges and CT cut-offs for the *SMN1* and *RPP30* analytes, 3200 de-identified DBS with unknown genotypes were obtained from the Alberta Newborn Screening lab. The reference range for the SMA assay was determined by using the mean ± 1.96 × standard deviation (SD) and calculated to be 23.91–28.03, although cut-offs were extended to flag values 18 > CT > 30 as the final screen positive cut-off. The reference range for the *RPP30* assay was determined by using the mean ± 1.96 × SD and calculated to be 22.87–26.06, although cut-offs were extended to flag values 18 > CT > 28.

### 3.2. Analytical Validation and Evaluation of the qPCR Assays

The SMA multiplex qPCR assay underwent a thorough validation process following established internal guidelines. During the validation, various parameters were evaluated to assess the performance and reliability of the assay. These parameters included analytical sensitivity and specificity, limit of blank, repeatability, intermediate precision, reproducibility, and robustness. By rigorously evaluating these parameters, the assay’s effectiveness in detecting *SMN1* was assessed, ensuring its suitability for screening purposes (Table 1).

- Analytical sensitivity and specificity: the assay was able to correctly identify the tested de-identified previously genotyped control samples, including 19 homozygous absence of exon 7 and 35 normal controls, which were comprised of 20 samples with heterozygous absence of exon 7 and 15 samples with 2 copies of *SMN1* (Supplementary Table S2). The data show that the assay analytical specificity and sensitivity are 100%, which makes it suitable for our screening purposes (Table 2).

- Repeatability: The assessment of intra-run repeatability involved comparing 14 de-identified DBS samples and 10 controls with known *SMN1* copy numbers in quadruplicate within a single run (Supplementary Table S3). All samples were correctly identified in all of the quadruple runs with 100% intra-run concordance, demonstrating the good repeatability of both *SMN1* and the *RPP30* assays. The intra-assay coefficient of variability (%CV) for *SMN1* CT values ranged from 0.54% to 3.71%, with an average of 0.65%. The %CV for *RPP30* CT values ranged from 2.0% to 4.9%, with an average of 0.71%. These values demonstrate the acceptable repeatability of the assay.
- Intermediate precision: The assessment of inter-run repeatability was performed by two technologists testing the same 96 de-identified DBS samples and the control samples with known *SMN1* copy numbers (Supplementary Table S3). For all the runs 100% of agreement between runs was observed and the CT values were in the expected ranges. The inter-assay %CV for *SMN1* CT values ranged from 0.65% to 6.51%, with an average of 2.39%. The %CV for *RPP30* CT values ranged from 0.68% to 4.1%, with an average of 1.89%. These values demonstrate the acceptable inter-run repeatability of the assay.
- Reproducibility: To monitor interlaboratory reproducibility and conduct proficiency testing, 10 External Quality Assessment (EQA) samples with known *SMN1* copy numbers, specifically designed for SMA analysis, were analyzed. The results of this analysis can be found in Supplementary Table S3. The analysis revealed that all of the samples were identified correctly, indicating a 100% agreement in interlaboratory concordance.
- Robustness: The robustness of the assay was evaluated by comparing the impact of master mix age on the CT values. The experiment involved running the same de-identified DBS samples while storing the master mix at 4 °C. The results indicated that the master mix can be stored at 4 °C for up to 10 days without significantly affecting the CT values (Supplementary Table S3).

These validation results highlight the accuracy and reliability of the designed SMA multiplex assay, making it perfect for our screening purposes.

### 3.3. Alberta Pilot Project

Screening outcomes: A one-year pilot project was conducted to screen a total of 47,005 babies born between February 2022 and February 2023 in Alberta for SMA using our validated multiplex qPCR assay. During the first year, six samples tested positive for SMA. Each of these six positive samples was repeated in duplicate the following day, and all confirmed the initial test results. Upon detection of each positive screen, a genetic counsellor contacted the ordering provider and a pediatric neurologist who then contacted the infant’s family. Additional blood samples were collected from the infants for confirmatory testing. Five of the six screen positive samples were confirmed to have SMA. The diagnostic test, which was performed using an MLPA kit, confirmed the homozygous absence of exon 7 of *SMN1*. Additionally, the test determined that all five of the screen positive cases in our study had three copies of *SMN2* (Supplementary Table S4). The re-evaluation of the SMA assay’s performance at the end of the first year of SMA newborn screening indicated that the analytical sensitivity of this screening multiplex assay remained at 100% and the analytical specificity was determined to be 99.999%.

### 3.4. Clinical Outcomes and Treatment

**Figure 2:**
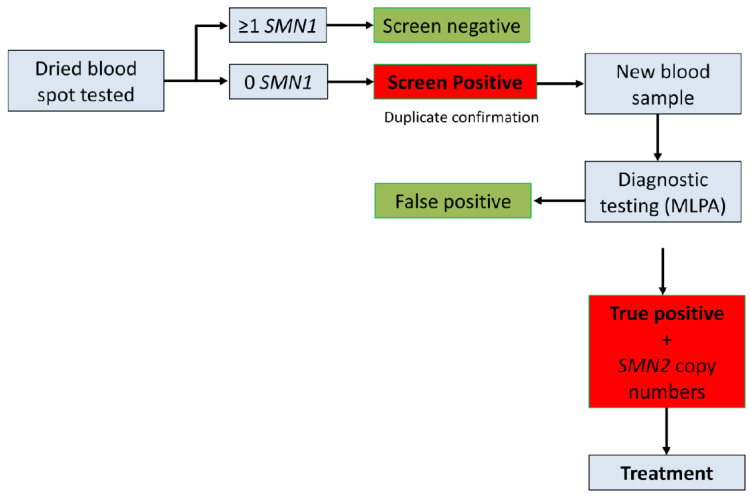
Timeline–diagnosis algorithm established in Alberta Newborn Screening Program. All DBS samples collected from across the province and received at the newborn laboratory in Edmonton undergo screening for SMA at the Molecular Genetics Lab (MGL). All samples with zero-copy of the *SMN1* gene are subjected to duplicate testing, and labeled as screen positive only if both tests yield positive results. This process typically requires 6–8 days. Upon obtaining a positive screening result, a genetic counselor immediately contacts the pediatric neurologist, who arranges a meeting with the proband’s family to collect new blood samples for confirmatory diagnostic testing. The diagnostic confirmation test is conducted on the new blood samples from the proband, using the MLPA kit at MGL. This test not only confirms the results of the screening test but also determines the copy number of the *SMN2* gene. On average, this diagnostic testing process takes 6 to 10 business days. Treatment is selected based on the copy numbers of *SMN2* and is initiated as soon as possible.

The screening results were released at 6–8 days of age, and an additional 6–10 business days on average were required for confirmatory testing. This enabled us to establish a definitive diagnosis between 13 to 27 days (Figure 2). All five confirmed cases had three copies of *SMN2* and were eligible to receive either onasemnogene abeparvovec-xioi or nusinersen based on criteria established by Alberta Health [22,23]. The application process and drug procurement for these SMA DMTs requires a minimum of 12 to 14 days. Four out of five infants who were asymptomatic at the time of treatment received onasemnogene abeparvovec-xioi between 25 to 30 days after birth (Table 3). Treatments were delayed for one patient (Case 2) due to initial high maternally transferred AAV9 neutralizing antibodies (NAb) and parental concerns for nusinersen as it requires intrathecal injections; this infant received risdiplam at 71 days of life after developing areflexia and sparse tongue fasciculations, followed by *SMN1* gene replacement therapy at 111 days of life when the Nab result returned negative (Table 3). All five infants remain well and have made excellent developmental progress after receiving treatment.

## 4. Discussion

We successfully developed and validated a multiplex qPCR SMA assay for NBS purposes. This was achieved by incorporating the SMA screening reagents into the existing SCID qPCR NBS assay, aiming to maximize cost-effectiveness. Over the course of a one-year pilot project, we screened 47,005 newborn babies for a homozygous absence of exon 7 in the *SMN1* gene. We identified six newborns with homozygous absence of exon 7 in *SMN1*, and five of them were confirmed by MLPA diagnostic testing. Evaluation of the assay’s performance during the pilot year indicated that it is accurate and robust, with no identified technical problems. The analytical sensitivity and specificity were determined to be 100% and 99.999%, respectively, which is considered ideal for a NBS program. These results indicate that the birth prevalence of SMA in Alberta is 1 in 9401 (95% CI [1 in 8618, 1:10,343]), which is within the range of frequencies published in other recent studies [24,25]. Interestingly, Kernohan and colleagues reported their first year of NBS for SMA from the province of Ontario [26]. A total of 139,900 infants were tested, and five infants were confirmed positive for SMA cases, with an estimated birth prevalence of 1 in 27,960 for Ontario. The observed variability in incidence rates may be attributed to population composition, as previous studies have indicated significant differences in the frequency of SMA carriers among various ethnicities [24,27].

All the identified SMA cases in our study had three copies of *SMN2*. While this is a relatively small number of cases, this finding is not inconsistent with our clinical experience or other reports that observed the majority of SMA patients having three copies of *SMN2*. In Ontario, Canada, five cases of SMA were identified in the first year of the Newborn screening program, two had two copies of *SMN2*, three had three copies and one had four copies [26]. Similarly, newborn screening programs in Germany and Belgium have reported a majority of positive cases with three or more copies of *SMN2* [28,29]. Some SMA cohort studies have also consistently shown a higher proportion of cases with three copies of *SMN2* compared with those with two copies [4,30,31,32]. For example, Calucho et al. reported that 43% of 625 Spanish SMA patients had two copies of *SMN2*, while 46% had three copies [4]. They also compiled data from publications spanning 1999–2018, reporting a worldwide distribution of 32% with two *SMN2* copies and 48% with three copies in a total of 2834 cases. Additionally, in a ten-year period prior to the introduction of the newborn screening program, 31% and 45% of patients diagnosed with SMA in Alberta had two and three copies of *SMN2*, respectively (unpublished data). Previous general population studies have also demonstrated a strong inverse relationship between the copy number of *SMN1* and *SMN2* [33,34]. Given that gene conversion from *SMN1* to *SMN2* is one of the main mechanisms in *SMN1* loss, it can be hypothesized that such gene conversion would lead to a decrease in *SMN1* copy number and an increase in *SMN2* copy numbers among SMA carriers and patients. Finally, considering that the population we screened was small, it is possible that the observed results could be random. Further investigation is required to elucidate the population composition of Alberta and the distribution of *SMN1* and *SMN2* copy numbers in the general population.

In approximately 96% of patients affected by SMA, biallelic *SMN1* deletion and/or gene conversion constitutes the underlying molecular cause. In approximately 4% of patients with SMA, other types of mutations that result in *SMN1* loss of function can be observed, where they occur with an *SMN1* deletion in a compound heterozygous state. The multiplex qPCR assay has been specifically designed to identify *SMN1* deletion and gene conversion cases, rendering it incapable of detecting other potentially causative types of mutations such as missense mutations, including c.731C>T p.(Pro244Leu) and c.332C>G p.(Ala11Gly), as well as frameshift mutations like c.431delC p.(Pro144Glnfs*5), which have been reported in several patients [35]. Given that the incidence rate of SMA in Alberta in the first year of NBS was approximately 1:10,000 and there are approximately 50,000 newborns in the province each year, we anticipate missing one patient with SMA every five years. This issue can only be resolved by implementing the newly developed long read sequencing, which enables us to identify not only the gene deletion but also all different types of mutations that can result in loss of function.

### False Positive Case

For one newborn, the initial collection was misplaced during transport. The Alberta Health Newborn Screening Application generated a recollection order for the infant because screening results were not completed by 10 days of age. The second collection was received first at the laboratory at 13 days of age and yielded a normal *SMN1* copy number result. Subsequently, the initially misplaced sample arrived at the laboratory at 24 days of age. The cause of the transport delay and the storage conditions of the sample could not be evaluated by the laboratory. This sample resulted in an amplification curve upon testing, with a corresponding CT value exceeding our *SMN1* cut-off, and was considered positive, while the other two analytes (*RPP30* and TREC) were within the normal range (Supplementary Table S5). This outcome raised concerns and consequently, a third collection was requested, which yielded a normal result upon analysis. However, to prevent the possibility of overlooking a positive newborn case, this baby was reported as a positive screen. Subsequent diagnostic testing confirmed that the baby was a carrier for *SMN1* deletion, with three copies of *SMN2*. Further investigation into the reason behind the initial collection’s lack of proper *SMN1* amplification was not pursued.

## 5. Conclusions

We developed and validated a multiplex qPCR assay for NBS for SMA. During a one-year pilot project, we screened 47,005 newborns in Alberta and identified six SMA-positive cases, five of which were confirmed by diagnostic testing. Our results from the first year of SMA NBS indicate that this multiplex qPCR assay is straightforward, automated, and cost-effective. Additionally, the assay showed high sensitivity and specificity, making it very suitable for ongoing NBS. All five affected infants remain well and have made excellent developmental progress after receiving SMA-specific DMTs.

## Figures and Tables

**Table 1:** Parameters assessed during the validation process of the assay.

Assay Performance Parameters	
Population study	3200 de-identified DBS collected >4 years ago
Limit of blank	45 DBS interspersed with 45 filter blanks
Sensitivity, Specificity, Accuracy	20 positive, 20 carriers, and 15 normal DBS
Repeatability	24 known samples run in quadruple
Intermediate Precision	Same 96 DBS samples run in 4 different runs
Reproducibility	Proficiency samples tested
Robustness	Durability of prepared mastermix tested

**Table 2:** Validation of the SMA multiplex assay: determination of sensitivity, specificity, and accuracy. Fifty-four known 0, 1, 2 copy *SMN1* controls were tested using the SMA multiplex assay to evaluate its performance.

Genotype	Samples
True Negative	35
True Positive	19
False Negative	0
False Positive	0
Total valid	54
Sensitivity	100%
Specificity	100%
Accuracy	100%

**Table 3:** Timeline and outcome of confirmed cases during 1st year of Alberta SMA NBS program.

SMA Case	1	2	3	4	5
Age (in days) when NBS sample was collected	1	1	1	1	1
Age (in days) when sample was received age	3	1	2	2	3
Age (in days) when positive screen reported	8	7	6	6	7
Age (in days) when parents were contacted	8	7	6	6	7
Age (in days) when seen in clinic and confirmatory lab sent	9	9	7	8	7
Age (in days) when confirmatory results reported	17	27	15	15	13
*SMN1* copies	0	0	0	0	0
*SMN2* copies	3	3	3	3	3
Age (in days) when 1st treatment given	29	72	28	30	25
Age (in days) when 2nd treatment given	N/A	111	N/A	N/A	N/A
Symptomatic before 1st treatment? Yes/No	No	Yes	No	No	No

The Alberta newborn screening lab and the diagnostic lab conduct testing on a five-day-per-week basis.

## Data Availability

Data is contained within the article and Supplementary Material.

## Supplementary Materials

The following supporting information can be downloaded at: https://www.mdpi.com/article/10.3390/ijns9030042/s1.
