# Supplementary material for: Alberta Spinal Muscular Atrophy Newborn Screening—Results from Year 1 Pilot Project
Source: Int J Neonatal Screen. 2023 Jul 27;9(3):42. doi: 10.3390/ijns9030042 (PMC10443376; doi:10.3390/ijns9030042)
Supplement: Supplementary file 1 [file IJNS-09-00042-s001.zip › IJNS-2463316 supplementary tables S1,2,4,5.pdf]

Supplementary Materials

Table S1. Sequences of Primers and Probes Used in the Multiplex PCR Assay for Newborn Screening of SMA.

Primer/Probe	Oligo Sequence
TREC_F	5'- TGC TGA CAC CTC TGG TTT TTG TAA -3'
TREC_R	5'- GTG CCA GCT GCA GGG TTT AG -3'
TREC_Probe FAM	5'- /FAM/ ATG CAT AGG CAC CTG C /MGBNFQ -3'
RPP30_F	5'- AGA TTT GGA CCT GCG AGC G -3'
RPP30_R	5'- GAG CGG CTG TCT CCA CAA GT -3'
RNaseP_Probe VIC	5'- /VIC/ TTC TGA CCT GAA GGC TCT GCG CG /MGBNFQ -3'
SMN1_F	5'- CTT GTG AAA CAA AAT GCT TTT TAA CAT CCA T -3'
SMN1_R	5'- GAA TGT GAG CAC CTT CCT TCT TTT T -3'
SMN1_Probe ABY	5'- /ABY/ AGG +GTT +T+C+A +GAC /MGBNFQ/ -3'
SMN2_Blocker	5'- AG+G +GTT +T+T+A +GAC -3'

Table S2. List of controls used during the validation: *SMN1* copy number was determined using MLPA before being used for validation purposes.

Sample	SMN1 Copy Number	SMN2 Copy Number	Status
1001	0	3	Patient
1002	1	2	Carrier
1003	1	3	Carrier
1004	1	3	Carrier
1005	1	2	Carrier
1006	0	3	Patient
1007	1	2	Carrier
1008	0	2	Patient
1009	1	3	Carrier
1010	1	2	Carrier
1011	0	3	Patient
1012	0	4	Patient
1013	0	3	Patient
1014	0	2	Patient
1015	0	3	Patient
1016	0	3	Patient
1017	0	3	Patient
1018	0	3	Patient
1019	1	2	Carrier
1020	0	3	Patient
1021	1	2	Carrier
1022	1	3	Carrier
1023	0	4	Patient
1024	2	4	Normal
1025	1	1	Carrier
1026	1	3	Carrier
1027	1	1	Carrier
1028	0	2	Patient
1029	0	3	Patient
1030	1	3	Carrier
1031	0	4	Patient
1032	1	3	Carrier
1033	1	3	Carrier
1034	1	1	Carrier
1035	1	1	Carrier
1036	0	3	Patient
1037	2	1	Normal
1038	0	3	Patient
2001	0	3	Patient
2002	1	2	Carrier
2003	1	3	Carrier
2005	1	2	Carrier
2006	0	3	Patient
SMA Control 1	2	2	Normal
SMA Control 2	2	2	Normal
SMA Control 3	2	2	Normal
SMA Control 4	2	not determined	Normal
SMA Control 5	2	not determined	Normal
SMA Control 6	2	not determined	Normal
SMA Control 7	2	not determined	Normal
SMA Control 8	2	not determined	Normal
SMA Control 9	2	not determined	Normal
SMA Control 10	2	not determined	Normal
SMA Control 11	2	not determined	Normal
SMA Control 12	2	not determined	Normal
SMA Control 13	2	not determined	Normal

Table S4. The MLPA analysis of the 5 cases that screened positive showed a homozygous deletion of exon 7 in the *SMN1* gene. All of the cases had 3 copies of *SMN2*.

Sample	SMN1 exon 7 ratio	SMN2 exon 7 ratio	MLPA* results
Case 1	5.21	148.5	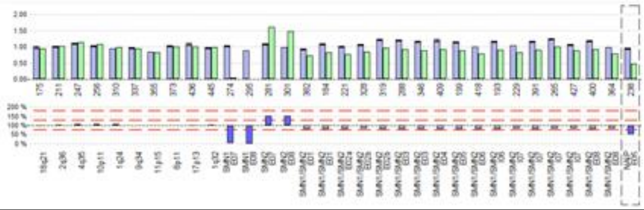
Case 2	0	145.09	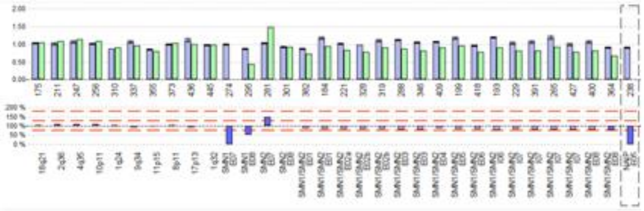
Case 3	5.73	143.43	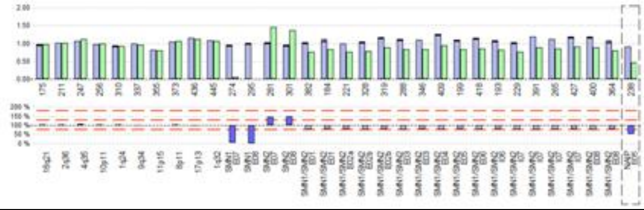
Case 4	0	144.89	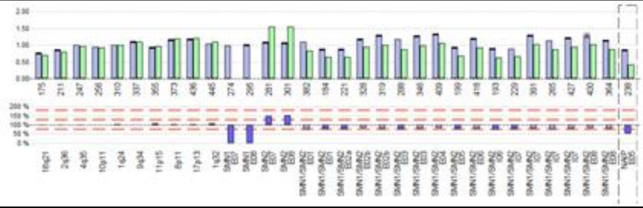
Case 5	8.89	144.43	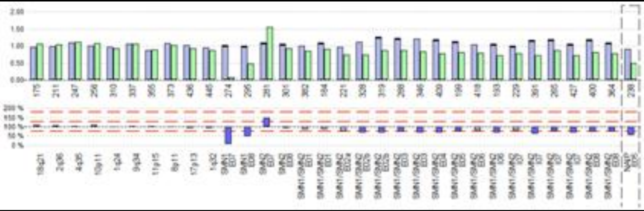

*MRC Holland Probemix P021 version B1; JSI Sequence Pilot MLPA module.

Table S5. CT values for obtained for the false positive in three separate collections.

	SMN1 CT	*RPP30* CT	TREC CT
Collection 1	undetermined	26.91	36.69
Collection 2	27.80	25.33	32.25
Collection 3	28.49	27.72	32.58
